# DeepKaryo-Check: a two-stage automated screening framework for chromosomal numerical and structural abnormalities in clinical karyotype analysis

**DOI:** 10.3389/fcell.2026.1836850

**Published:** 2026-07-01

**Authors:** Wenjing Li, Xiaoyan Liang, Hongjian Yu, Liang Sun

**Affiliations:** Binzhou People’s Hospital Affiliated to Shandong First Medical University/College of Medical Information and Artificial Intelligence, Shandong First Medical University and Shandong Academy of Medical Sciences, Jinan, Shandong, China

**Keywords:** chromosomal abnormalities, clinical karyotype analysis, clinical screening, case prioritization, deep learning, geometric normalization, proof-of-concept study

## Abstract

**Introduction:**

Chromosomal abnormalities, including numerical abnormalities such as aneuploidy and structural abnormalities such as microdeletions and complex rearrangements, remain challenging to detect in a stable and reliable manner using automated methods in clinical karyotype analysis. This difficulty is primarily attributable to substantial non-rigid chromosome deformation, variability in sample preparation and imaging conditions, and the persistent scarcity of abnormal cases in real-world clinical datasets, all of which hinder the construction of generalizable morphological representations. In clinical practice, limited sample size is a common condition rather than an exception, which further intensifies the trade-off between sensitivity and specificity in automated screening systems. Therefore, achieving robust high-specificity screening under small-sample conditions is a central problem in the automated analysis of chromosomal abnormalities. In this study, we focus on cytogenetic image analysis and propose a structured modeling framework designed for clinical screening scenarios. Recent advances in multi-omics and machine learning have enabled more comprehensive characterization of disease states. Although the present work primarily focuses on cytogenetic imaging, the proposed framework provides an extensible basis for future integration with heterogeneous biological data.

**Methods:**

We propose a multi-stage automated chromosomal analysis framework, termed *DeepKaryo-Check*, which integrates explicit geometric priors with consistency-based deep representation learning. The framework first exploits the relatively stable spatial organization of clinical karyotype images to perform geometry-guided anchoring of chromosomal instances and impose basic structural constraints, thereby reducing variability introduced by slide preparation and imaging. Individual chromosomes are then geometrically normalized and represented as one-dimensional width-profile sequences. On this basis, explicit difference modeling and multi-instance aggregation are employed to assess structural consistency between homologous chromosomes at the patient level, supporting the identification of fine-grained structural abnormalities such as microdeletions. All decision thresholds are determined exclusively through cross-validation within the training data, while the independent test set remains fully isolated prior to evaluation.

**Results:**

In the independent Stage I test set of 520 karyotype images, including 260 normal and 260 numerically abnormal images, DeepKaryo-Check achieved an accuracy of 95.19%. The framework correctly classified 249 of 260 normal images and 246 of 260 abnormal images. The independent Stage II test cohort included 45 patients, comprising 29 patients without structural abnormalities and 16 patients with structural abnormalities. At the locked high-specificity threshold, the patient-level ROC-AUC was 0.9978 and the PR-AUC was 0.9963. All 29 patients without structural abnormalities were correctly classified, while 13 of 16 patients with structural abnormalities were identified, corresponding to a sensitivity of 0.8125 and a specificity of 1.000. Three patients with structural abnormalities were missed, and no patient without a structural abnormality was classified as positive.

**Conclusion:**

These findings indicate that explicitly incorporating domain-relevant geometric priors into automated analysis workflows can improve the reliability of chromosomal structural abnormality screening under sample-limited conditions. *DeepKaryo-Check* is not intended to replace definitive clinical diagnosis, but rather to serve as a proof-of-concept screening and prioritization tool that supports early decision-making in automated karyotype analysis when data are constrained, providing methodological guidance for subsequent large-scale, multi-center validation studies.

## 1 Introduction

Karyotype analysis remains one of the most fundamental and irreplaceable techniques in clinical cytogenetics, and is widely used for the diagnosis of congenital genetic disorders, hematological malignancies, and infertility-related chromosomal abnormalities. Despite the rapid development of molecular diagnostic technologies, karyotype analysis continues to serve as one of the core reference methods in current clinical practice Amor and Gardner (2025); Hassold et al. (1980). This technique provides clinicians with a genome-wide overview of chromosomal architecture and plays an irreplaceable role in the diagnosis of complex structural abnormalities, such as balanced translocations, inversions, and low-level mosaicism.

Although next-generation sequencing and chromosomal microarray analysis provide advantages in resolution and throughput, they do not offer a complete overview of chromosome-level structural organization. The unique value of G-banded karyotype analysis lies in its ability to directly reveal the global structural organization of the genome within a single assay, which is crucial for the accurate diagnosis of structural abnormalities such as balanced translocations and inversions. Unlike these molecular approaches, karyotype analysis provides clinicians with a genome-wide structural perspective and thereby facilitates the detection of chromosomal structural abnormalities. This global view is particularly important for identifying balanced translocations, inversions, and low-level mosaicism, which may not be fully captured by existing molecular methods and therefore represent inherent diagnostic blind spots (Amor and Gardner, 2025; Wan, 2014). Accordingly, although molecular methods provide important technical advantages in specific contexts, karyotype analysis continues to play an irreplaceable role in practical clinical workflows, including cancer cytogenetics, prenatal diagnosis, and reproductive medicine, particularly for the assessment of structural abnormalities and as a stable reference for molecular testing.

Despite its irreplaceable role in the diagnostic framework, karyotype analysis in practice remains heavily dependent on expert manual interpretation and is highly labor-intensive. A complete evaluation of a single case typically requires experienced cytogeneticists to spend substantial time identifying and segmenting overlapping chromosomes from metaphase images, performing geometric corrections, and conducting detailed comparisons at the band and sub-band levels. Even under relatively standardized laboratory conditions, this process is difficult to fully disentangle from subjective judgment. Previous studies have reported that chromosome image selection, band classification, and the interpretation of structural abnormalities may vary substantially across operators and even between laboratories Zabawski et al. (2005); Quinn et al. (2023). Such variability undermines result consistency and complicates quality control as well as cross-center reproducibility.

This human-centered analytical paradigm creates throughput bottlenecks, increases uncertainty in diagnostic outcomes, and limits the scalability of karyotype analysis workflows. To address these challenges, automated and computer-aided analysis methods have attracted increasing clinical attention. In the context of karyotype analysis, automation is better positioned not as a complete replacement for expert judgment, but as a high-specificity screening and quality-control tool that supports clinicians in repetitive and labor-intensive tasks, such as preliminary screening and case prioritization. The goal of such systems is to provide stable and controllable technical assistance, reduce the false-positive burden, and preserve the final diagnostic decision for experienced clinical experts. This positioning is more consistent with the practical requirements of current karyotype analysis workflows and may improve overall diagnostic efficiency. Nevertheless, current automated systems still have limitations when dealing with complex or atypical cases. Future work should therefore further explore how to improve robustness in challenging clinical scenarios and gradually reduce dependence on manual expert assessment. This role does not weaken the clinical value of automation; rather, it aligns more closely with the realistic implementation of computer-aided diagnosis in contemporary clinical workflows (Guo et al., 2022).

In recent years, deep learning techniques have been widely adopted in medical image analysis and have also been introduced into chromosomal image processing tasks, including automated detection, classification, and banding pattern recognition Song et al. (2025); Kang et al. (2024); Li et al. (2021). Some studies simplify karyotype analysis into an end-to-end image classification problem, directly predicting chromosomal categories or abnormality types from single-chromosome images after prior segmentation and normalization. Under controlled experimental conditions, such approaches can achieve favorable classification performance Yang et al. (2023). However, these methods are typically built upon idealized input assumptions. Existing reviews have pointed out that they lack systematic modeling and constraints for factors frequently encountered in real clinical settings, such as chromosome overlap, non-rigid bending, and variability in slide preparation quality S and S S, (2025); Al et al. (2025). When these assumptions no longer hold, model performance often degrades rapidly, substantially limiting their generalizability in practical clinical workflows.

As automated karyotype analysis moves toward practical clinical deployment, the main challenge lies not simply in adopting increasingly complex model architectures, but in effectively exploiting the intrinsic structural information contained in karyotype data. In routine clinical practice, input images are usually arranged as standardized karyograms according to the International System for Human Cytogenomic Nomenclature (ISCN), rather than being provided as raw metaphase images. Therefore, the key problem is not to re-interpret these layouts from scratch, but to explicitly model the already standardized spatial organization as stable and controllable geometric priors, including chromosome relative positions, ordering rules, and homologous pairing relationships. Such priors can support downstream analysis without requiring additional pixel-level annotations.

Many existing methods overlook this premise. Some approaches directly treat karyograms as natural images and apply end-to-end deep learning models for classification, whereas others rely on pixel-level instance segmentation or Siamese networks for chromosome-level modeling (Wang et al., 2024; Arora and Dhir, 2016). Although these methods have achieved promising performance in specific settings, they often depend on idealized assumptions or additional annotation costs. Moreover, their robustness and interpretability remain limited when dealing with chromosome overlap, morphological distortion, and imaging variability, which restricts their long-term deployment in real-world clinical environments.

A more fundamental challenge arises from the extreme scarcity of structural abnormality cases in clinical datasets. Unlike numerical abnormalities, which are directly defined by changes in chromosome number, structural abnormalities often manifest as subtle band-level or sub-band-level morphological differences with small effect sizes and unstable appearances. Under highly imbalanced data distributions, these abnormal patterns can be easily overwhelmed by normal samples, leading to pronounced class bias during model training and making it difficult to learn reliable fine-grained discriminative representations (Japkowicz and Stephen, 2002; He and Garcia, 2009).

To address this issue, several studies have adopted Siamese or homologous-comparison architectures, reformulating structural abnormality detection as a problem of modeling differences between homologous chromosomes (Bechar et al., 2023; Li et al., 2024). These approaches have shown effectiveness on standardized datasets and are capable of capturing subtle chromosome-level structural differences. However, although such methods may perform stably under controlled data partitioning strategies, their robustness in real-world clinical datasets can still be affected by sample imbalance, abnormal-case scarcity, and heterogeneous imaging conditions. Therefore, Siamese or homologous-difference modeling is more appropriately regarded as a component of structural abnormality screening rather than a fully autonomous diagnostic system. By integrating patient-level decision making with homologous-difference modeling, *DeepKaryo-Check* aims to improve screening accuracy while accommodating the practical requirements and data limitations of clinical cytogenetic workflows.

Based on the above considerations, we propose an integrated two-stage automated karyotype analysis framework, named *DeepKaryo-Check*, which aims to achieve high-specificity chromosomal abnormality screening without altering existing slide preparation procedures or clinical review workflows. In the first stage, the standardized spatial organization of karyotype images is exploited, and geometric constraints are introduced to enable robust chromosome layout parsing, chromosome counting, and quality control. In the second stage, homologous chromosome pairs are constructed after geometric normalization. Explicit difference modeling and an attention-based multiple instance learning model, referred to as *HomNet-V3*, are then used to perform patient-level consistency assessment, thereby enhancing the screening capability for structural abnormalities and improving stability under data-limited conditions.

The main contributions of this study are summarized as follows:

1. We propose a geometric modeling strategy based on the standardized spatial structure of karyotype images. This strategy enables robust chromosome layout parsing and counting without requiring pixel-level annotations, while also providing clinically meaningful quality-control information.
2. To address the scarcity of structural abnormality samples and the severe class imbalance commonly observed in clinical datasets, we propose a structural abnormality screening method based on homologous-difference modeling and patient-level decision making, improving stability and reliability under small-sample conditions.
3. We construct a clinical workflow-oriented two-stage automated analysis framework that integrates numerical abnormality screening and structural abnormality assessment. The framework is designed to support high-specificity screening and case prioritization, rather than to replace final clinical diagnosis.

## 2 Materials and methods

### 2.1 Study design and data source

This retrospective, single-center methodological study was designed to develop and evaluate DeepKaryo-Check as a high-specificity automated screening and quality-control framework for routine clinical cytogenetic workflows. The study used de-identified digital karyotype images obtained from routine clinical cytogenetic examinations at Binzhou People’s Hospital Affiliated to Shandong First Medical University. The analyzed images represented the standard clinical outputs of karyotype examination, rather than raw metaphase microscopy images. In routine practice, chromosomes had been arranged into conventional homologous-pair layouts by clinical cytogenetic personnel, and cytogenetic findings were reported according to the International System for Human Cytogenomic Nomenclature (ISCN).

Clinical cytogenetic interpretations served as the reference standard for model development and evaluation. All karyotype images were interpreted and arranged during routine diagnostic procedures by experienced clinical cytogeneticists according to institutional clinical practice. No additional image acquisition procedures or experimental pixel-level annotation processes were introduced for this study. Before analysis, all data were fully de-identified and contained no information that could be used to identify individual patients. The study protocol was approved by the Medical Research Ethics Committee of Binzhou People’s Hospital, and the requirement for informed consent was waived because of the retrospective use of de-identified clinical images.

The initial cohort comprised digital karyotype images from 461 patients, including 376 patients clinically interpreted as having normal karyotypes and 85 patients diagnosed with chromosomal abnormalities. Each image was linked to a single patient, and multiple karyotype images could be available for the same patient. Patient-level normal or abnormal labels were assigned according to the corresponding clinical cytogenetic diagnostic reports. The dataset included both normal and abnormal karyotype cases and was used as a clinically relevant proof-of-concept dataset for evaluating automated screening under limited and imbalanced clinical data conditions.

DeepKaryo-Check was designed as a screening and case-prioritization tool rather than a replacement for definitive manual karyotype diagnosis. Its outputs were intended to provide structured reference information for cytogenetic experts, support preliminary screening and quality control, and help prioritize potentially abnormal cases for expert review. The final diagnostic decision remained with qualified clinical specialists.

According to the logic of clinical karyotype interpretation, the automated analysis was divided into two complementary stages operating at different decision levels. Stage I focused on image-level screening for numerical chromosomal abnormalities using the complete digital karyogram. This stage incorporated geometry-guided chromosome localization, standardized layout mapping, chromosome counting, and quality-control procedures. Stage II focused on patient-level screening for structural chromosomal abnormalities by analyzing homologous chromosome pairs from the same patient after geometric normalization. This staged design was intended to approximate the clinical review process, progressing from global assessment of chromosome number to local comparison of homologous chromosome morphology.

The Stage I numerical abnormality screening module was constructed using 376 patients with normal karyotypes and 58 patients with numerical chromosomal abnormalities, comprising 1,622 normal karyotype images and 260 abnormal karyotype images. The detailed distribution of numerical chromosomal abnormalities is summarized in Table 1. Stage I performance was evaluated at the image level.

**TABLE 1:** Distribution of numerical chromosomal abnormalities in the Stage I abnormal dataset.

Numerical chromosomal abnormality	Number of images
Trisomy 21/Down syndrome	114
Klinefelter syndrome	39
Trisomy 18/Edwards syndrome	33
XYY syndrome	29
Triple X syndrome	15
Turner syndrome	14
Trisomy 13/Patau syndrome	10
Triploidy	6
**Total**	**260**

Bold values indicate the total number of images.

The Stage II structural abnormality screening module was developed and evaluated using a representative patient-level subcohort constructed for homologous-chromosome consistency assessment. This subcohort included 108 patients without structural abnormalities and 27 patients with chromosomal structural abnormalities. Homologous chromosome pairs were used as analytical instances, resulting in 5,738 normal instances and 149 abnormal instances. The detailed classification of structural abnormalities is summarized in Table 2. All experiments involving structural abnormalities followed strict patient-level data partitioning, ensuring that data from the same patient did not appear across training, validation, and independent test subsets.

**TABLE 2:** Distribution of structural chromosomal abnormalities in the Stage II dataset.

Structural chromosomal abnormality	Number of instances
Translocation	114
Isochromosome of the long arm of the X chromosome, *i*(Xq)	10
Deletion	20
Duplication	5
**Total**	**149**

Bold values indicate the total number of instances.

Numerical abnormality screening and structural abnormality screening were treated as independent but complementary analytical tasks, with separate data construction and evaluation protocols. For structural abnormality screening, all decision thresholds were determined exclusively through cross-validation within the training data and were kept fixed during subsequent testing. The independent test set was not used for model development, hyperparameter tuning, or threshold selection.

### 2.2 Overall two-stage screening framework

**FIGURE 1:**
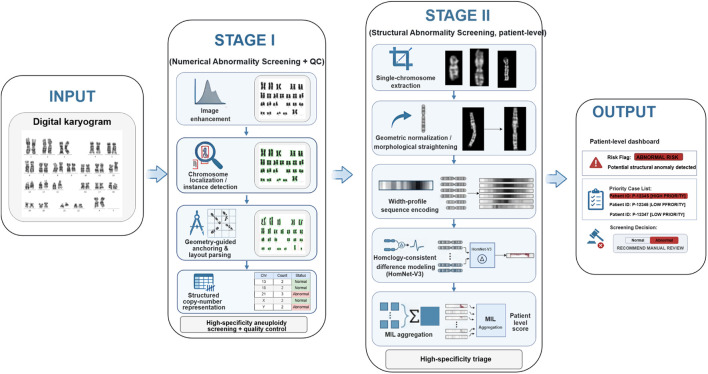
Overview of the *DeepKaryo-Check* framework. The framework follows a coarse-to-fine cascaded design to support automated screening and quality control in routine clinical cytogenetic workflows. Stage I: Aneuploidy screening. The Segment Anything Model (SAM) is first used to identify potential chromosome regions, reducing the influence of imaging noise and local morphological variation. A YOLO model is then applied for chromosome instance detection and localization. The detected chromosome instances are organized through geometry-guided anchoring to maintain spatial consistency and homologous-pairing relationships, and are mapped onto a standardized karyotype layout space for numerical abnormality screening. Rule-based post-processing is further used to refine the detection results and perform quality control, ensuring the accuracy and reliability of the final screening output. Stage II: Structural abnormality screening. Homologous chromosome pairs are geometrically normalized, and each chromosome instance is transformed into a one-dimensional width-profile sequence. The HomNet-V3 model is then used for homologous-consistency modeling, in which bidirectional cross-attention aligns homologous chromosomes and quantifies their structural differences. Finally, multiple instance learning aggregates difference features from multiple cells to generate a patient-level risk score for structural chromosomal abnormalities.

DeepKaryo-Check adopts a coarse-to-fine two-stage cascaded analysis strategy to support automated screening and quality control in routine clinical cytogenetic workflows, as shown in (Figure 1). Unlike end-to-end approaches based on a single complex model, the proposed framework decomposes sources of uncertainty into sequential processing steps across different stages and spatial scales, introducing stage-specific constraints and decision logic at each level. This design reflects the clinical logic of karyotype review, in which cytogeneticists first assess global chromosome number and layout quality and then perform more detailed comparison of homologous chromosomes when structural abnormalities are suspected.

**FIGURE 2:**
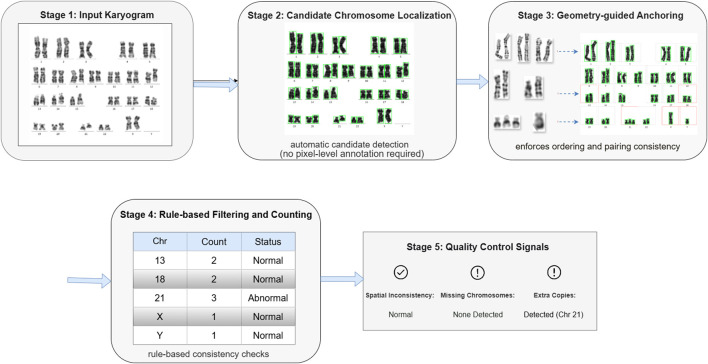
Geometry-guided aneuploidy screening and quality control pipeline in Stage I. Stage I operates on complete digital karyograms and follows a multi-step, geometry-guided workflow. Candidate chromosome regions are automatically detected and then organized through geometry-guided anchoring within a standardized karyotype layout. Rule-based filtering and counting are subsequently applied to derive structured copy-number representations. The pipeline also generates interpretable quality-control signals, including spatial inconsistency, missing chromosomes, and extra copies, to support manual review and downstream structural abnormality analysis.

Stage I takes complete digital karyograms as input, with the primary objective of obtaining stable and interpretable chromosome localization and copy-number information to support reliable screening of chromosomal numerical abnormalities. Rather than relying on fine-grained pixel-level manual annotations, this stage integrates image enhancement, automated instance detection, and geometry-guided anchoring mechanisms to organize and constrain detected chromosomal instances, which are subsequently mapped into a standardized karyotype layout space (Figure 2). Within this space, chromosomes are localized, ordered, and counted, yielding a consistent structured representation that supports screening for aneuploidies and other numerical abnormalities.

From a system design perspective, Stage I is treated as an independent module for numerical abnormality screening and quality control. Its outputs are not only used to indicate potential numerical abnormalities, but also explicitly expose issues such as localization shifts, fragmented detections, or inconsistencies in homologous pairing. These quality control signals provide a reliable pairing foundation and essential reference information for subsequent structural abnormality analysis.

Following chromosome instance localization and homologous pairing, Stage II performs patient-level modeling for structural abnormality risk assessment. Rather than treating individual chromosomes or single cells as independent decision units, this stage jointly analyzes multiple homologous chromosome pairs from the same patient, reflecting the clinical practice of assessing global consistency and repeated evidence during karyotype review. Stage II explicitly models structural differences between homologous chromosomes, with a particular focus on subtle morphological inconsistencies at the band and sub-band levels, which are often attenuated in global feature aggregation frameworks.

**FIGURE 3:**
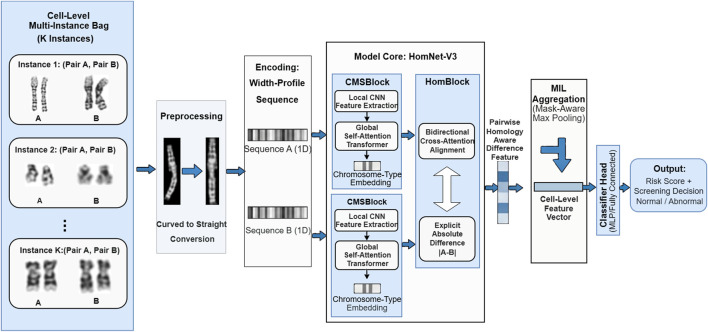
Architecture of HomNet-V3 for structural abnormality screening. Homologous chromosome pairs within each cell are geometrically normalized, encoded as width-profile sequences, and processed by the CMSBlock and HomBlock to obtain pairwise homology-aware difference features. Valid pair-level features are aggregated within each cell using mask-aware max pooling and classified to generate a cell-level abnormality probability. The patient-level score is defined as the maximum probability across all available cells from the same patient and is used to produce the final normal/abnormal screening decision.

Specifically, Stage II employs a homology-difference modeling network with physical consistency constraints (HomNet-V3) to quantitatively characterize structural differences between homologous chromosome pairs (Figure 3). Multiple homologous difference representations derived from the same patient are aggregated at the patient level using a multi-instance learning strategy, and final screening decisions are made under a high-specificity constraint Ilse et al. (2018). The evaluation protocol for this stage is consistent with the patient-level data partitioning and threshold-locking strategy adopted in the experimental design, ensuring that screening behavior remains statistically and operationally controllable.

The two stages therefore operate at different but complementary decision levels. Stage I provides image-level chromosome counting and quality-control information for numerical abnormality screening, whereas Stage II provides patient-level structural abnormality risk assessment based on homologous chromosome comparison. The framework was designed to support clinical screening, quality control, and case prioritization, rather than to replace definitive manual karyotype diagnosis.

In this study, the design and implementation of the proposed framework were based on the following technical platforms and tools. Python was used as the primary programming language for framework development, and PyTorch was adopted as the deep learning framework for model training and optimization. For chromosome instance localization and copy-number arrangement, we used the YOLOv8 model, a member of the You Only Look Once family, to perform object detection. The model automatically detected chromosome instance locations and bounding boxes and generated candidate regions for subsequent analysis. YOLOv8 enabled efficient and accurate chromosome localization, providing the basis for downstream copy-number arrangement and structural assessment. For data processing and image preprocessing, the OpenCV library was used for grayscale conversion, binarization, and feature extraction. To reduce image noise and morphological variation, Gaussian smoothing and interpolation methods were applied to improve image consistency and comparability. All training and evaluation procedures were performed on CUDA-enabled GPU devices to accelerate computation. In addition, datasets and intermediate results were stored in Pickle format, and custom scripts were developed for data preprocessing, normalization, and formatting. The integration of these technical platforms and tools ensured the efficiency and reliability of the proposed framework when processing complex cytogenetic image data.

### 2.3 Stage I: geometry-guided aneuploidy screening

In routine clinical karyotype analysis, the identification of chromosomal numerical abnormalities relies on stable recognition and accurate counting of chromosome instances within complete karyograms. However, under real slide preparation and imaging conditions, uneven background illumination, chromosome curvature, close proximity, and partial occlusion are common, often resulting in blurred chromosome boundaries. These factors increase the burden of manual counting and introduce uncertainty. The design objective of Stage I is therefore to provide stable, interpretable, and verifiable chromosome counting results under such conditions, supporting reliable screening of numerical abnormalities while establishing a robust quality control foundation for subsequent structural abnormality analysis S and S S, (2025).

Unlike approaches that primarily optimize detection accuracy at the level of individual chromosome instances, this stage emphasizes the consistency of overall counting behavior across varying imaging conditions. Accordingly, the method does not depend on fine-grained pixel-level manual annotations. Instead, a series of engineering-oriented constraints are introduced to organize detected chromosome instances into a spatial structure that conforms to karyotype analysis logic, thereby reducing the influence of local detection errors on final counting decisions Wang et al. (2024); Al et al. (2025).

In practice, input digital karyogram images are first subjected to unified contrast and detail enhancement to address common issues such as insufficient contrast and background non-uniformity. This step aims to improve the discriminability of chromosome bodies at the image level rather than to achieve precise boundary delineation. By suppressing background responses while preserving the continuity of chromosome contours, more stable input conditions are provided for subsequent instance localization S and S S, (2025); Wang et al. (2024).

Based on the enhanced images, the system performs automated localization of potential chromosome regions within the karyogram. Given the high cost and limited reusability of pixel-level manual annotations in karyotype analysis, the detection model is trained using automatically generated candidate annotations as supervisory signals. Specifically, a general-purpose segmentation model (Segment Anything Model, SAM) is first applied to obtain preliminary segmentation results and candidate chromosome regions for downstream instance localization and rule-based organization Kirillov et al. (2023). These candidate regions are then used to generate proxy annotations for training a target detection model (YOLO). Subsequently, candidate detections are regularized using morphological and spatial priors derived from karyotype analysis. This process is not intended to produce strictly accurate boundaries, but rather to ensure that each chromosome can be consistently identified and distinguished in terms of spatial position and overall morphology.

After chromosome instance detection, the candidate results were not directly used to determine numerical abnormalities. Instead, all detected chromosome instances were mapped into a standardized karyotype layout space and matched against predefined physical and organizational constraints. To achieve this, we used the YOLO model as the chromosome localization platform to automatically detect the position and bounding box of each chromosome instance. The YOLO outputs were then processed through a post-processing stage for geometric correction of chromosome instances. Specifically, within the standardized layout space, relative position, ordering, and copy-number relationships were explicitly constrained to ensure that local pixel-level detection errors, fragmented instances, or localization offsets would not directly affect the final counting results. This process was performed automatically without manual intervention. Under predefined physical and organizational constraints, the model corrected the detected chromosome instances through further comparison and ordering, ensuring consistency with the standard karyotype layout pattern. Functionally, this geometry-guided constraint procedure served as a structured correction process applied to the detection results, aligning the automated analysis with the global layout-based workflow used in karyotype interpretation. In this way, local errors in chromosome instance localization could be effectively reduced, ensuring that the relative position and ordering of each chromosome pair in the layout space were properly maintained. Subsequent correction of localization errors and instance deduplication, such as fragment merging, were also completed by the automated workflow to optimize chromosome copy-number arrangement.

Within the overall system, Stage I is designed and evaluated as an independent module for chromosomal numerical abnormality screening and quality control. Its outputs are not only used to indicate aneuploidies and other numerical abnormalities, but also to explicitly reveal potential issues such as localization anomalies, fragmented detections, or inconsistencies in homologous pairing. These signals provide reliable reference information for manual review and establish a robust pairing foundation for downstream structural abnormality risk assessment. The module was intended to assist expert review by providing reproducible counting and quality-control information and was not intended to replace definitive interpretation by clinical cytogeneticists.

### 2.4 Stage II: homology-consistent structural abnormality screening

Stage II was designed for patient-level screening of chromosomal structural abnormalities. In contrast to numerical abnormalities, which can primarily be assessed through chromosome counts, structural abnormalities may present as localized differences in chromosome length, contour, centromeric configuration, or banding-related morphology. These differences may be subtle and can be confounded by non-pathological chromosome curvature, variable orientation, uneven staining, and image-acquisition variability. Stage II therefore compared homologous chromosomes after geometric normalization and combined information across chromosome pairs and cells to generate a patient-level structural abnormality screening score.

Clinical interpretation of chromosomal structural abnormalities commonly involves comparison between homologous chromosomes and review of repeated observations from the same patient. Accordingly, individual chromosomes and individual cells were not treated as independent final patient-level decision units. Homologous chromosome pairs served as analytical instances, multiple instances from the same cell formed a cell-level bag, and information from all available cells was subsequently aggregated to obtain the patient-level screening score Li et al. (2024).

**FIGURE 4:**
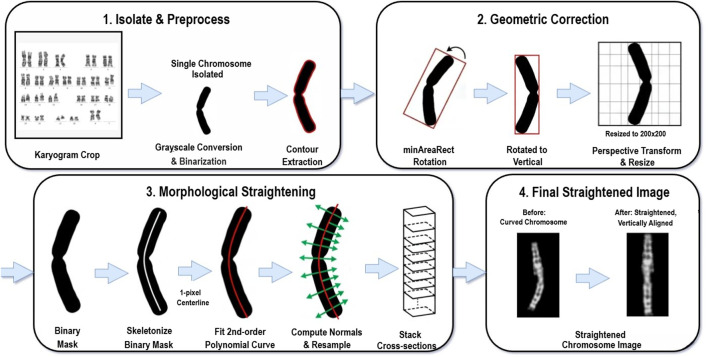
Geometric normalization and morphological straightening of individual chromosome instances. This figure illustrates the step-by-step preprocessing pipeline used to reduce non-pathological morphological variability prior to structural abnormality analysis. First, individual chromosome regions are isolated from the karyogram, followed by grayscale conversion, binarization, and contour extraction. Next, geometric correction is applied by estimating the minimum-area bounding rectangle, rotating chromosomes to a vertical orientation, and performing perspective transformation and resizing to a unified resolution. Subsequently, morphological straightening is conducted by skeletonizing the binary mask, fitting a low-order polynomial curve to the chromosome centerline, and resampling cross-sections along the estimated normals to correct physiological curvature. The final output is a vertically aligned, straightened chromosome image that preserves longitudinal band information while enabling consistent comparison across different chromosome instances.

Before structural abnormality analysis, non-pathological morphological variations introduced during slide preparation and imaging should be minimized as much as possible. To this end, a unified geometric normalization procedure was applied to each chromosome instance to place chromosomes into a comparable representation, specifically by normalizing their orientation, scale, and longitudinal structure. First, individual chromosome regions were extracted from the karyotype image, and obvious background noise was removed. The principal axis of each chromosome was then corrected, followed by scale normalization. On this basis, morphological correction was performed through skeleton extraction and centerline fitting to reduce the influence of non-pathological bending on longitudinal morphological alignment, as illustrated in Figure 4. This process was implemented using image-processing tools from the OpenCV library, including thresholding, morphological operations, and interpolation, to ensure geometric alignment of chromosome instances and to reduce potential errors introduced during image acquisition and slide preparation. To further alleviate local misalignment and positional offsets, a customized morphological straightening algorithm was used. Specifically, the longitudinal centerline of each chromosome was estimated and smoothed, and the chromosome morphology was then standardized along this centerline. Finally, Gaussian smoothing and interpolation were applied to standardize chromosome features to a uniform scale. Through these procedures, chromosome instances from different sources became geometrically comparable, providing a consistent basis for subsequent structural difference assessment.

Within the unified geometric representation space, each chromosome was further encoded as paired longitudinal width-profile sequences describing changes in chromosome width along its longitudinal axis. This representation was derived from the foreground contour geometry of each chromosome and preserved the longitudinal characteristics of the left and right chromosome boundaries while reducing the influence of isolated pixel-level noise. Through resampling, normalization, and smoothing, a length-standardized sequential representation was obtained, enabling homologous chromosome morphology to be compared on a common scale.

The feature construction procedure consisted of the following steps:

1. Image reading and preprocessing. For each chromosome image, the grayscale image was first extracted, and the foreground chromosome region was identified by thresholding. Binary segmentation was used to separate the chromosome region from the background. Based on the foreground mask, the longitudinal structural information was obtained by scanning the chromosome along the vertical axis.
2. Feature extraction. A row-wise scanning strategy was used to extract the left and right width profiles of each chromosome. For each row along the normalized chromosome axis, foreground pixels were identified and divided relative to the estimated central position. The left and right transverse extents of the chromosome foreground were then recorded to form row-level width features. The resulting paired sequences represented longitudinal changes in chromosome contour and width.
3. Interpolation and smoothing. Because different chromosomes may have different longitudinal resolutions, tthe extracted profiles were resampled to a fixed sequence length of 100 sampling points. This ensured that all chromosome instances had the same feature length. Gaussian smoothing was then applied to the extracted profile features to reduce the influence of local noise and small non-pathological fluctuations.
4. Feature normalization. To ensure comparability across chromosome instances, the extracted left and right width profiles were normalized to the range of 0 to 1. Light Gaussian smoothing was applied to reduce isolated pixel-level fluctuations while preserving the overall longitudinal contour and width patterns.
5. Data structure and storage. After preprocessing, feature extraction, interpolation, smoothing, and normalization, the width-profile feature of each chromosome was stored as a two-channel array with a shape of (2,100) , where the first dimension represented the left and right profiles and the second dimension represented the 100 sampled points along the longitudinal axis. Each chromosome was therefore represented as a two-channel array with a shape of (2, 100), corresponding to the left and right width-profile sequences. Two chromosomes from the same homologous pair were combined to form an input instance with a shape of (2, 2, 100) for homologous-difference modeling.

On this basis, we introduced a homologous-consistency modeling network, termed *HomNet-V3*, to quantitatively characterize structural differences between homologous chromosome pairs. Instead of performing “normal/abnormal” classification on individual chromosomes, the model uses the relative differences between paired homologous chromosomes as the primary discriminative signal. To account for slight misalignment or local displacement that may occur during slide preparation and image acquisition, explicit alignment and difference-computation mechanisms were incorporated into the comparison process, thereby reducing false responses caused by non-pathological variations (Li et al., 2024).

HomNet-V3 was designed to characterize morphological differences between paired homologous chromosomes rather than to classify individual chromosomes independently. For each chromosome, the paired width-profile sequences were encoded using one-dimensional convolutional operations to capture local longitudinal morphological patterns, while multi-head self-attention was used to integrate information across distant positions along the chromosome axis. Chromosome-type embeddings were incorporated to provide information on the expected chromosome category. The resulting representations of the two homologous chromosomes were then aligned bidirectionally and compared through explicit absolute-difference modeling. The difference representations from both comparison directions were compressed and combined to generate a 128-dimensional homology-aware feature for each chromosome pair. Within each cell, the valid pair-level features were aggregated using mask-aware max pooling to obtain a cell-level feature vector. This cell-level representation was subsequently passed through a lightweight fully connected classification head, which reduced the feature dimension from 128 to 64 and then generated two logits corresponding to the normal and structurally abnormal screening classes at the cell level.

By integrating these modules, *HomNet-V3* enables effective structural abnormality screening from chromosome profile representations. In particular, it is designed to capture and quantify subtle morphological differences between homologous chromosome pairs, making it suitable for structural abnormality screening under limited-sample and clinically heterogeneous conditions.

By integrating these components, HomNet-V3 generated homology-aware difference representations from paired chromosome width-profile sequences. These representations were intended to characterize longitudinal differences in chromosome contour and width between homologous chromosomes. They were not intended to provide a complete representation of grayscale G-banding texture or all cytogenetically relevant structural features.

When multiple cells were available for the same patient, the patient-level structural abnormality score was defined as the maximum cell-level abnormality probability across all available cells from that patient. The implemented workflow therefore included two analytical levels: feature aggregation across homologous chromosome-pair instances within each cell, followed by probability aggregation across cells at the patient level. The continuous patient-level score was converted into a normal/abnormal screening decision using the fixed high-specificity operating threshold described in Section 2.5.

The Stage II output was intended to support structural abnormality screening, case prioritization, and expert review rather than definitive cytogenetic diagnosis. All final clinical interpretations remained based on review by qualified cytogenetic specialists and the established clinical reference standard (Guo et al. 2022).

### 2.5 Model training and decision strategy

To achieve stable and controllable screening behavior under real clinical data conditions, the structural abnormality screening model adopts a pretraining–fine-tuning strategy that combines representation pretraining with clinical fine-tuning under patient-level data partitioning. Rather than directly optimizing clinical detection performance, this design aims to first establish stable and reproducible homology-difference response patterns under the practical constraint of extreme scarcity of structural abnormality samples Japkowicz and Stephen (2002); He and Garcia (2009), and subsequently calibrate patient-level decision behavior using a limited number of real clinical cases.

During the pretraining stage, the model is trained on a large set of normal samples together with synthetically generated difference samples. The primary purpose of this stage is to encourage the model to learn consistent and transferable representations of homologous structural differences Li et al. (2024). Training and validation accuracy and loss were monitored to assess optimization convergence and the stability of representation learning. It should be emphasized that evaluation at this stage is solely intended to verify the stability of the representation learning process and does not directly reflect performance on real clinical structural abnormality detection tasks.

Following pretraining, the model is initialized with the pretrained parameters and fine-tuned on real clinical case data at the patient level. Only real patient data are used at this stage, and no synthetic abnormal samples are introduced, in order to avoid biasing final screening behavior through artificially constructed differences. Both training and evaluation are conducted with patients as the minimum decision unit, in accordance with the case-centered interpretation logic of clinical karyotype analysis Ilse et al. (2018). To prevent potential data leakage, patient-stratified cross-validation is employed during fine-tuning, ensuring that samples from the same patient do not appear simultaneously in the training and validation sets.

Screening decision thresholds are determined according to a high-specificity–first principle. Threshold selection is performed exclusively within the training data by scanning candidate operating points on the validation set. Among thresholds that satisfy a predefined specificity constraint, the threshold yielding the highest sensitivity is selected for each training fold. To obtain stable and reproducible screening behavior, the median of thresholds obtained across cross-validation folds is adopted as a fixed operating threshold and remains unchanged during subsequent independent testing. This strategy is designed to avoid over-optimization to a single data split and more closely reflects real clinical deployment scenarios, in which decision thresholds are typically fixed once defined Guo et al. (2022).

Overall, the training and decision strategy employed in this study does not aim to maximize single-run evaluation metrics. Instead, it emphasizes consistency, controllability, and interpretability of screening behavior across different case compositions and data partitions. By using pretraining to stabilize homology-difference representations and introducing a high-specificity–constrained threshold-locking mechanism during patient-level fine-tuning, the proposed approach is better suited as an auxiliary screening and quality control tool, providing reliable support for structural abnormality risk assessment in real clinical environments.

## 3 Results

### 3.1 Patients’ cohorts

Based on the two-stage analysis framework described above, chromosomal numerical abnormality screening (Stage I) and structural abnormality screening (Stage II) were evaluated independently. The two tasks were designed with separate data construction schemes, evaluation protocols, and decision levels to avoid data mixing and to ensure that performance assessments for each stage remained task-specific.

The patient cohort for Stage I numerical abnormality screening was constructed from the overall dataset and included both normal karyotypes and common aneuploidy types, with complete karyotype images serving as the basic analysis unit. This stage comprised karyotype images from 376 patients with clinically normal karyotypes and 58 patients with numerical abnormalities, totaling 1,622 normal karyotype images and 260 abnormal karyotype images.

For data partitioning in Stage I, 1,090 normal karyotype images were used for model training and 272 normal images were reserved for validation. The remaining 260 normal karyotype images, together with the 260 abnormal karyotype images, formed an independent test set of 520 images for the final evaluation of aneuploidy screening performance. This test set was not involved in any stage of model training or parameter selection.

The patient cohort for Stage II structural abnormality screening was constructed from the overall dataset under strict patient-level independence. This cohort included 108 patients without structural abnormalities and 27 patients with chromosomal structural abnormalities, and was used for model training, validation, and independent evaluation. In this stage, homologous chromosome pairs were used as analysis instances, yielding 5,738 normal instances and 149 abnormal instances. Among them, 29 patients without structural abnormalities and 16 patients with chromosomal structural abnormalities were used as the independent test set.

In terms of data organization, each patient could contribute multiple cell samples, and each cell sample formed a variable-length instance set (cell-level bag) consisting of all homologous chromosome pairs within that cell. Patient-level clinical labels were used during fine-tuning. Cell-level bags served as the basic sampling units for batch updates, whereas threshold selection and performance evaluation were conducted at the patient level. For each patient, the structural abnormality score was defined as the maximum cell-level abnormality probability across all available cells from that patient.

Cohort partitioning followed a predefined patient-level split into training and test sets, with no patient overlap between the two. Within the training cohort, patient-stratified cross-validation was further applied to support model selection and to assess the stability of operating thresholds. The final operating threshold used for testing was determined as the median of thresholds obtained across cross-validation folds and was evaluated only once on the independent test cohort, thereby ensuring the independence and reproducibility of the reported results.

### 3.2 Performance of stage I: aneuploidy screening and quality control

**FIGURE 5:**
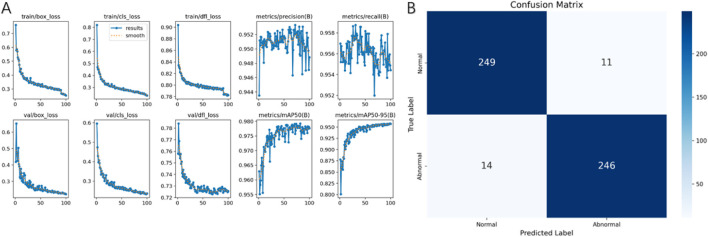
Performance evaluation of the Stage I numerical abnormality screening module. **(A)** Training and validation curves of the chromosome detection model. The box loss, classification loss, and distribution focal loss showed gradual convergence during training, while the detection metrics, including precision, recall, mAP50, and mAP50–95, remained stable on the validation set, indicating stable optimization without obvious overfitting. **(B)** Confusion matrix of Stage I screening on the independent test set, including 260 normal karyotype images and 260 aneuploid karyotype images. The model correctly classified 249 normal images and 246 abnormal images, with 11 false-positive and 14 false-negative cases, demonstrating stable screening performance for numerical chromosomal abnormalities.

The Stage I module was evaluated independently as a numerical abnormality screening and quality-control component. Its performance evaluation did not focus on the recognition of complex structural abnormalities, but rather on the stability of chromosome counting results and the reliability of abnormality indication. To assess the robustness of the detection component before downstream geometry-guided constraints and rule-based post-processing, the detection model was monitored separately during training and validation. Figure 5A summarizes the convergence behavior of the loss functions and the trends of major detection metrics on the training and validation sets. All loss terms showed a gradual decreasing trend and converged during the later training stages, while the validation metrics closely followed the training metrics. No obvious overfitting or performance degradation was observed, indicating stable optimization of the detection module.

In the independent test set, 260 normal karyotype images and 260 aneuploid karyotype images were evaluated. The test cohort covered common types of numerical abnormalities, including trisomy 21, trisomy 18, and sex chromosome abnormalities, and was not involved in any model training or parameter selection process. The corresponding image-level confusion matrix is shown in Figure 5B, and the quantitative results are summarized in Table 3. On the independent test set, the Stage I module demonstrated stable screening performance in terms of accuracy, sensitivity, and specificity. A high proportion of abnormal cases were correctly identified, while the false-positive rate among normal samples remained low. These results are consistent with the intended role of Stage I as a preliminary aneuploidy screening module, which aims to preserve screening sensitivity while avoiding excessive and unnecessary abnormality alerts.

**TABLE 3:** Quantitative performance of Stage I aneuploidy screening on the independent test set.

Metric	Value (%)	Clinical significance
Accuracy	95.19	Overall ability of the system to correctly classify all samples
Sensitivity	94.62	Ability to correctly identify abnormal cases; high sensitivity indicates a low false-negative rate
Specificity	95.77	Ability to correctly identify normal cases; high specificity indicates a low false-positive rate

**FIGURE 6:**
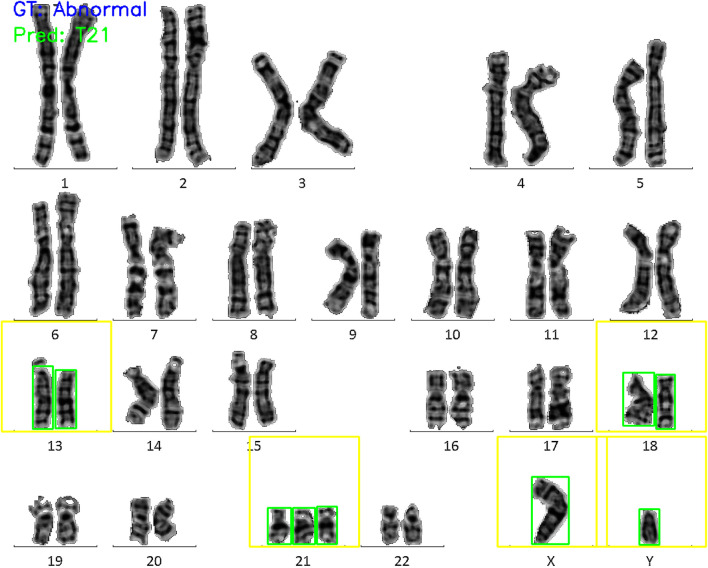
Qualitative visualization of Stage I aneuploidy screening on a representative trisomy 21 case. The figure shows the processing result of a karyogram with a ground-truth abnormal label, which is correctly identified by the system as trisomy 21. Detected chromosome instances are organized within a standardized karyotype layout, where geometry-guided anchoring enables consistent localization and copy-number counting. The presence of three copies of chromosome 21 is clearly highlighted, illustrating how the proposed Stage I module supports interpretable numerical abnormality indication and facilitates manual verification.

To further illustrate the operational mechanism of Stage I, a representative aneuploidy case was selected for qualitative visualization. Figure 6 shows the processing result of a trisomy 21 karyotype image. The system localized and counted chromosome regions within the standardized karyotype layout, illustrating how geometry-guided anchoring and counting rules were used to indicate the numerical abnormality and provide reference information for expert review. In addition, a comparative analysis was performed to investigate the screening behavior under different post-processing configurations, as shown in Table 4. The results showed that when only the basic detection model was used, the screening performance was relatively lower across all three metrics. After introducing rule-based candidate-box filtering, the overall screening performance improved substantially. When fragment deduplication was further incorporated, the performance remained stable without additional degradation. These results indicate that the standardized post-processing workflow contributes to consistent numerical abnormality screening behavior, rather than relying solely on the detection model itself.

**TABLE 4:** Comparison of Stage I screening performance under different post-processing configurations.

Model configuration	Key components	Accuracy (%)	Sensitivity (%)	Specificity (%)
Model A (base model)	YOLOv8	93.65	93.85	93.46
Model B (base + filtering)	+ filter_yolo_boxes	95.19	94.62	95.77
Model C (full model)	+ deduplicate_fragments	95.19	94.62	95.77

### 3.3 Performance of Stage II: patient-level structural abnormality screening

**FIGURE 7:**
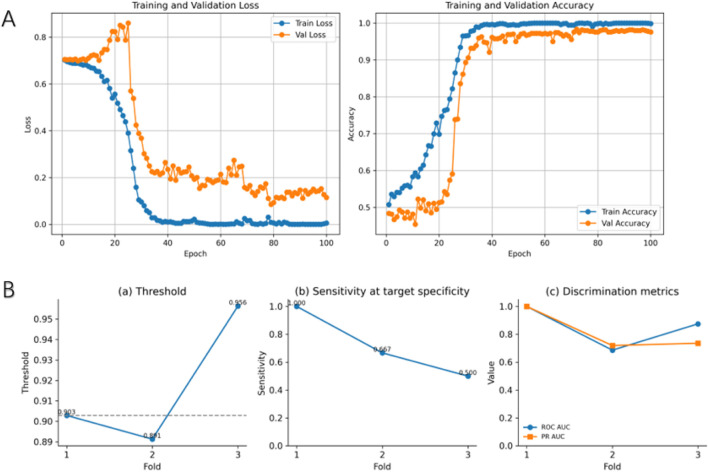
Pretraining and cross-validation evaluation of the Stage II structural abnormality screening module. **(A)** Training and validation loss curves during the pretraining phase across 100 epochs. The training and validation losses showed consistent decreasing trends and stable convergence, indicating that the homologous-difference representation was effectively optimized without obvious divergence. **(B)** Training and validation accuracy curves across epochs. Both curves exhibited stable and consistent performance, suggesting effective learning and generalization during pretraining. (c) Threshold selection and patient-level performance evaluated by three-fold cross-validation. (a) Decision thresholds determined from patient-level validation predictions in each fold. The median threshold of 0.90 was selected as the fixed operational threshold for subsequent evaluation. (b) Sensitivity at the target specificity across folds, showing stable abnormal-case detection under the high-specificity constraint. (c) Discrimination performance across folds, including ROC-AUC and PR-AUC. Both metrics remained high across folds, demonstrating robust patient-level discrimination between normal and structurally abnormal cases.

The Stage II module evaluated structural abnormality screening using geometrically normalized homologous chromosome pairs. Performance was assessed at the patient level, with particular attention to discrimination and operation at the prespecified high-specificity threshold under class imbalance. During model development, the convergence behavior in the pretraining stage was first examined. Using normal samples and synthetically generated difference samples, the training and validation curves showed similar convergence trends, as shown in Figure 7A. It should be noted that the purpose of this stage was to verify the stability of homologous-difference representation learning, rather than to directly reflect model performance on real clinical structural abnormality cases.

After pretraining, the model was fine-tuned using real clinical case data at the patient level. A patient-stratified three-fold cross-validation strategy was adopted to ensure that samples from the same patient did not appear simultaneously in the training and validation sets. In each fold, the decision threshold was determined based on patient-level validation predictions. Despite variation across folds, the model performance remained consistent, with ROC-AUC and PR-AUC values maintained at relatively high levels on the validation sets, while the number of false-positive cases remained limited, as shown in Figure 7B.

**FIGURE 8:**
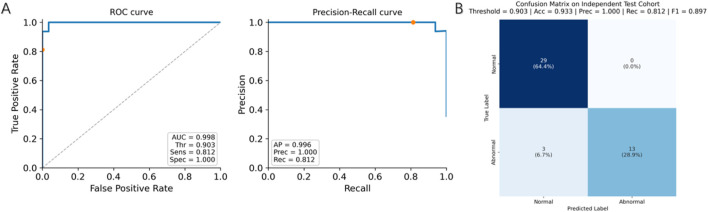
Independent patient-level evaluation of the Stage II structural abnormality screening module. **(A)** ROC curve and precision–recall curve on the independent evaluation cohort. The model achieved a ROC-AUC of 0.9978 and a PR-AUC of 0.9963, indicating strong discrimination between normal and structurally abnormal patients. The operating point was determined using the fixed threshold of 0.9029 derived from cross-validation. **(B)** Confusion matrix at the fixed operating threshold. Among 29 normal patients, all were correctly classified as normal, resulting in no false-positive cases. Among 16 patients with structural abnormalities, 13 were correctly identified as abnormal and 3 were missed. The corresponding accuracy, precision, recall, and F1-score were 0.933, 1.000, 0.812, and 0.897, respectively.

To obtain a unified and reproducible screening criterion, the median threshold derived from the three cross-validation folds was adopted as the fixed operating threshold and was kept unchanged in subsequent evaluation. Under this fixed-threshold setting, patient-level screening was performed on an independent evaluation cohort consisting of 29 normal patients and 16 patients with chromosomal structural abnormalities. None of these patients had been used in model training, cross-validation, or threshold selection. As shown in Figure 8, the model correctly classified all normal patients as normal, resulting in no false-positive cases. Among the structural abnormality cases, 13 patients were correctly identified as abnormal, while 3 patients were missed. The corresponding ROC-AUC and PR-AUC values reached 0.9978 and 0.9963, respectively, indicating strong patient-level discrimination between normal and structurally abnormal cases. At the fixed threshold of 0.9029, the model achieved an accuracy of 0.933, precision of 1.000, recall of 0.812, and F1-score of 0.897. These results demonstrate that the proposed method can maintain a high-specificity operating profile under imbalanced data conditions while effectively prioritizing most structural abnormality cases for further manual review.

**FIGURE 9:**
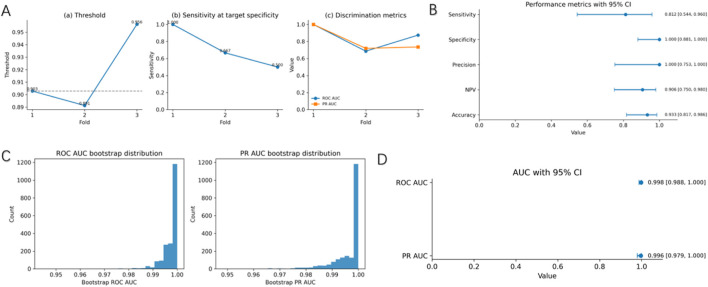
Statistical evaluation of the Stage II structural abnormality screening module across cross-validation folds and bootstrap analysis. **(A)** Performance across three-fold cross-validation. **(a)** Decision threshold values for each fold, with the horizontal dashed line indicating the fixed threshold used for final evaluation. **(b)** Sensitivity at the target specificity for each fold, showing moderate variation while maintaining stable abnormal-case detection. **(c)** Discrimination metrics across folds, including ROC-AUC and PR-AUC. Both metrics remained consistently high, indicating robust patient-level discrimination. **(B)** Confusion-matrix-derived performance metrics with 95% confidence intervals for the independent test cohort, including sensitivity, specificity, precision, negative predictive value, and accuracy. These results quantify the uncertainty of binary screening metrics under imbalanced data conditions. **(C)** Bootstrap distributions of AUC-based performance metrics. **(a)** Bootstrap distribution of ROC-AUC estimates, with most values concentrated near 1.00, indicating strong discrimination. **(b)** Bootstrap distribution of PR-AUC estimates, showing similarly high performance across bootstrap resampling. **(D)** ROC-AUC and PR-AUC estimates with corresponding 95% confidence intervals, further supporting the robustness and reliability of the proposed model in independent patient-level evaluation.

In Stage II, we further enhanced the evaluation protocol by incorporating statistical analyses to improve the reliability and interpretability of model assessment. First, 95% Clopper–Pearson confidence intervals were calculated for binary classification proportion metrics, including sensitivity, specificity, and precision. These intervals provided statistical uncertainty ranges for each metric, which is particularly important under severely imbalanced data conditions. Next, patient-level bootstrap 95% confidence intervals were calculated for ROC-AUC and PR-AUC to quantify the stability and generalizability of model evaluation, as shown in Figure 9C. In addition, cross-validation metrics across folds, including the decision threshold, sensitivity, specificity, ROC-AUC, and PR-AUC, were statistically summarized to verify the consistency of the model across different data subsets, as shown in Figures 9A,B. Based on these enhanced evaluation procedures, we reported the 95% confidence intervals of ROC-AUC and PR-AUC, confusion-matrix-derived metrics with their 95% confidence intervals, and cross-validation stability measures, including threshold, sensitivity, and AUC-related metrics, as shown in Figure 9D. These results indicate that, despite the imbalanced data distribution, the proposed method was able to distinguish patients with normal karyotypes from those with structural abnormalities while maintaining high specificity.

In the Stage II evaluation, HomNet-V3 was compared with a Siamese model and a direct inference model without clinical fine-tuning. The evaluated measures included the target specificity, operating threshold, ROC-AUC, and PR-AUC. Within the evaluated dataset, HomNet-V3 achieved the highest ROC-AUC and PR-AUC values among the three models. The Siamese model yielded lower ROC-AUC and PR-AUC values than HomNet-V3, whereas the direct inference model without clinical fine-tuning showed lower discrimination metrics than the fine-tuned models. The complete comparison is summarized in Table 5. ROC-AUC and PR-AUC, indicating limited discriminative ability without adaptation to the clinical structural abnormality dataset.

**TABLE 5:** Performance comparison of different models for Stage II structural abnormality screening.

Model	Target specificity	Threshold	Test ROC-AUC	Test PR-AUC
*HomNet-V3*	0.99	0.9029	0.9978	0.9963
Siamese model	0.99	0.9029	0.9698	0.9641
Direct inference without fine-tuning	1.00	0.5000	0.7522	0.6707

## 4 Discussion

Automated chromosomal karyotype analysis in real clinical environments has long been constrained by two fundamental factors. On the one hand, chromosomes exhibit pronounced non-rigid deformations during slide preparation and imaging, resulting in substantial morphological variability. On the other hand, structural abnormalities are intrinsically rare in clinical data, leading to persistent limitations in the number of samples available for model training and validation. Under these conditions, approaches that rely solely on end-to-end learning or continuously increasing model complexity often struggle to achieve stable and reproducible screening behavior. In light of this reality, the present study does not attempt to construct a fully automated diagnostic system covering all aspects of karyotype analysis, but instead focuses on screening stability and procedural controllability.

Experimental results indicate that separating numerical abnormality screening and structural abnormality screening into distinct stages helps mitigate the impact of uncertainty arising at different scales. In the first stage, explicit geometric constraints are introduced to organize and count chromosome instances, constraining detection results within a spatial structure consistent with karyotype analysis logic. The resulting copy number estimates are more stable across varying imaging conditions and are easier to verify during manual review. Compared with end-to-end segmentation approaches that heavily rely on pixel-level annotations S and S S, (2025), this strategy reduces dependence on annotation precision and better matches the data acquisition conditions encountered in real clinical practice.

During the structural abnormality screening stage, the analytical focus shifts from absolute classification of individual chromosomes to consistency assessment between homologous chromosome pairs Bechar et al. (2023); Li et al. (2024). The results suggest that, under patient-level decision settings, relative difference modeling leads to more consistent screening behavior. Many structural abnormalities manifest as localized disruptions at the band or sub-band level. Such changes are easily obscured by noise when analyzed using single-instance or global feature representations, but become more apparent under homologous comparison. By incorporating geometric normalization and physical consistency constraints, the proposed system is able to suppress instability induced by pose variation or spurious local differences, even under limited sample conditions.

From an application perspective, the screening characteristics observed at high-specificity operating points carry clear clinical relevance. Given the low prevalence of structural abnormalities, excessive false-positive rates can substantially increase manual review burden and undermine the practical utility of automated systems. In this study, the proposed framework maintains relatively conservative abnormality flagging behavior under fixed decision thresholds, making it more suitable as a triage and quality-control tool rather than for definitive diagnosis Guo et al. (2022). Cases flagged as abnormal can be prioritized for expert review, while cases without abnormality indications can be treated as reference information within routine workflows.

In addition, the one-dimensional width-profile features used in this study mainly reflect morphological variations and local width differences along the longitudinal axis of chromosomes. This representation is suitable for relative comparison between homologous chromosomes, but it cannot fully capture the complete complexity of chromosomal structure. Some structural abnormalities may be primarily manifested as G-banding texture changes, grayscale intensity distributions, local band deletions or duplications, centromere position shifts, or alterations in arm ratios, which are difficult to sufficiently represent using width profiles alone. Chromosome bending, stretching, uneven banding quality, and slide-preparation differences may also affect the stability of width-profile representations. Therefore, in this study, the width profile is more appropriately regarded as an interpretable morphological screening cue rather than a complete structural representation. Future studies may further incorporate raw image texture, G-banding grayscale intensity sequences, centerline curvature, arm ratio, centromere position, and deep visual features to improve the model’s ability to recognize complex structural abnormalities and subtle sub-band-level changes.

It should be noted that, under a high-specificity-oriented configuration, sensitivity to structural abnormalities remains constrained by data conditions. Current results indicate that the proposed approach is effective in detecting abnormalities with pronounced structural disruption, whereas subtle sub-band-level microdeletions or complex rearrangements with ambiguous breakpoints may still be missed. This observation reflects inherent limitations of structural abnormality screening based solely on morphological and grayscale banding information, and further underscores the role of the system as an assistive tool for expert interpretation rather than an independently operating diagnostic system.

Several limitations of this study should be acknowledged. The evaluation of structural abnormality screening was performed on a relatively limited preliminary dataset, which did not fully cover the diversity of complex structural abnormalities encountered in clinical practice. The synthetic abnormalities introduced during pretraining were mainly used to stabilize homologous-difference representation learning; however, their morphological complexity may not be fully equivalent to that of real clinical abnormalities. In addition, although model comparisons and independent test evaluations were conducted within the available dataset, this study still lacks external validation and comparison using large-scale public datasets. This is mainly because currently available public karyotype datasets usually lack complete patient-level labels, standardized homologous chromosome pairing information, clearly defined structural abnormality categories, and case-level annotations consistent with clinical diagnostic reports. Therefore, these datasets are difficult to directly use for the patient-level structural abnormality screening evaluation required in this study. The proposed two-stage framework has also not yet been jointly optimized in an end-to-end manner, and localization errors from Stage I may propagate to the subsequent structural screening module. Future work will expand the scale and diversity of real abnormal cases, conduct multicenter and public-dataset validation, and incorporate expert feedback mechanisms to support system deployment in real clinical environments. Meanwhile, we will further explore the integration of genomic and transcriptomic data to improve diagnostic accuracy and biological interpretability.

## 5 Conclusion

This study developed DeepKaryo-Check, a two-stage automated screening framework for chromosomal numerical and structural abnormalities in clinical digital karyograms. The framework combines geometry-guided chromosome localization and counting for numerical abnormality screening with homologous chromosome comparison and patient-level aggregation for structural abnormality assessment. By integrating structured geometric information with patient-level decision making, DeepKaryo-Check provides a workflow-compatible approach to automated karyotype screening under limited and imbalanced clinical data conditions.

DeepKaryo-Check is intended to support quality control, preliminary screening, and prioritization of cases for expert review rather than to replace definitive cytogenetic interpretation. The findings provide proof-of-concept evidence for its potential clinical utility, while further evaluation in larger, independent, and multicenter cohorts is needed to assess its generalizability and performance in prospective clinical workflows.

## Data Availability

The datasets generated and/or analyzed during the current study are not publicly available due to ethical and privacy restrictions related to clinical patient data, but are available from the corresponding authors upon reasonable request and with appropriate institutional approval.

## References

[B1] Al R. Ramesh B. R. K N. T R M. (2025). Automated system for chromosome karyotyping. 2025 AI-Driven Smart Healthc. Soc. 5 (0), 236–240. 10.1109/IEEECONF64992.2025.10962892

[B2] Amor D. J. Gardner R. M. (2025). Gardner and Sutherland’s Chromosome Abnormalities and Genetic Counseling. Oxford University Press. 10.1093/med/9780197747728.001.0001

[B3] Arora T. Dhir R. (2016). A review of metaphase chromosome image selection techniques for automatic karyotype generation. Med. & Biol. Eng. & Comput. 54, 1147–1157. 10.1007/s11517-015-1419-z 26676686

[B4] Bechar M. E. A. Guyader J.-M. El Bouz M. Douet-Guilbert N. Al Falou A. Troadec M.-B. (2023). Highly performing automatic detection of structural chromosomal abnormalities using siamese architecture. J. Mol. Biol. 435, 168045. 10.1016/j.jmb.2023.168045 36906061

[B5] Guo Z. Xie J. Wan Y. Zhang M. Qiao L. Yu J. (2022). A review of the current state of the computer-aided diagnosis (cad) systems for breast cancer diagnosis. Open Life Sci. 17, 1600–1611. 10.1515/biol-2022-0517 36561500 PMC9743193

[B6] Hassold T. Chen N. Funkhouser J. Jooss T. Manuel B. Matsuura J. (1980). A cytogenetic study of 1000 spontaneous abortions. Ann. Hum. Genet. 44, 151–164. 10.1111/j.1469-1809.1980.tb00955.x 7316468

[B7] He H. Garcia E. A. (2009). Learning from imbalanced data. IEEE Trans. Knowl. Data Eng. 21, 1263–1284. 10.1109/TKDE.2008.239

[B8] Ilse M. Tomczak J. M. Welling M. (2018). Attention-based deep multiple instance learning. arXiv preprint arXiv:1802.04712.

[B9] Japkowicz N. Stephen S. (2002). The class imbalance problem: a systematic study1. Intell. Data Anal. 6, 429–449. 10.3233/IDA-2002-6504

[B10] Kang S. Han J. Lee I. Joo H. Chung Y. Yang S. (2024). Chromosome analysis method based on deep learning: counting chromosomes and detecting abnormal chromosomes. Biomed. Signal Process. Control 91, 105891. 10.1016/j.bspc.2023.105891

[B11] Kirillov A. Mintun E. Ravi N. Mao H. Rolland C. Gustafson L. (2023). Segment anything. arXiv:2304.02643.

[B12] Li J. Chen B. Sun X. Feng T. Zhang Y. (2021). Banded chromosome images recognition based on dense convolutional network with segmental recalibration. Sheng Wu Yi Xue Gong Cheng Xue Za Zhi 38, 122–130. 10.7507/1001-5515.201912029 33899436 PMC10307562

[B13] Li J. Fu F. Wei R. Sun Y. Lai Z. Song N. (2024). “Chromosomal structural abnormality diagnosis by homologous similarity,” in Proceedings of the 30th ACM SIGKDD Conference on Knowledge Discovery and Data Mining (New York, NY: Association for Computing Machinery), 5317–5328. 10.1145/3637528.3671642

[B14] Quinn L. Tryposkiadis K. Deeks J. De Vet H. C. W. Mallett S. Mokkink L. B. (2023). Interobserver variability studies in diagnostic imaging: a methodological systematic review. Br. Journal Radiology 96, 20220972. 10.1259/bjr.20220972 37399082 PMC10392644

[B15] S S. D. S S V. C. (2025). “Segmentation of overlapping chromosomes using kriging based u-net,” in 2025 6th International Conference on Control, Communication and Computing (ICCC), 1–6. 10.1109/ICCC64910.2025.11077188

[B16] Song G. Su L. Wang X. Huang Z. Wang S. Fu Q. (2025). Deep learning-based chromosome segmentation and extraction: a comprehensive review of methodologies, challenges, and future directions. Neurocomputing 652, 131060. 10.1016/j.neucom.2025.131060

[B17] Wan T. S. K. (2014). Cancer cytogenetics: methodology revisited. Ann. Laboratory Med. 34, 413–425. 10.3343/alm.2014.34.6.413 25368816 PMC4215412

[B18] Wang C. Yu L. Su J. Shen J. Selis V. Yang C. (2024). Fully automatic karyotyping deep convolutional neural networks. IEEE Access 12, 46081–46092. 10.1109/ACCESS.2024.3380829

[B19] Yang C. Li T. Dong Q. Zhao Y. (2023). Chromosome classification via deep learning and its application to patients with structural abnormalities of chromosomes. Med. Eng. & Phys. 121, 104064. 10.1016/j.medengphy.2023.104064 37985030

[B20] Zabawski J. Wiktor A. Sikora M. Van Dyke D. L. (2005). Use reference bands to accurately estimate iscn band levels 400, 550, and 850. J. Assoc. Genet. Technol. 31 (9—13), 9–13. 15829739

