# Supplementary material for: DeepKaryo-Check: a two-stage automated screening framework for chromosomal numerical and structural abnormalities in clinical karyotype analysis
Source: Front Cell Dev Biol. 2026 Jul 1;14:1836850. doi: 10.3389/fcell.2026.1836850 (PMC13368874; doi:10.3389/fcell.2026.1836850)
Supplement: Supplementary file 1 [file DataSheet1.pdf]

Supplementary Material

1 DATA AUGMENTATION ALGORITHM

Below is the pseudocode describing the core algorithm used for data augmentation:

Algorithm 1: Data Augmentation Algorithm

```
Input: Input image Img
Output: Enhanced image final_img

Step 1: Dehaze the image;
if Img is grayscale then
    Convert Img to RGB;

Calculate the dark channel min_channel;
Apply erosion to obtain the dark channel dark_channel;
Estimate atmospheric light A using the brightest 0.1% pixels;
Calculate the transmission map t using the normalized dark channel norm_dark;
Compute t = 1 − ω × norm_dark;
Ensure t ≥ t0;
Restore image J using the dehazing formula;
Return the dehazed image;

Step 2: Denoise and sharpen the image;
Apply bilateral filtering for denoising to obtain denoised;
Convert Img to grayscale image gray;
Apply Laplacian sharpening to obtain sharpened;
Return the sharpened image;

Step 3: Convert to SAM-compatible format;
Convert the grayscale image to RGB format and obtain final_img;

Step 4: Batch processing;
foreach image in the input directory do
    Read the image;
    Apply dehazing, denoising, and sharpening procedures;
    Save the processed image to the output directory;
```

2 TRAINING ALGORITHM

Below is the pseudocode describing the training process for the YOLO model:

Algorithm 2: YOLO Model Training Algorithm

Input: Model path `MODEL_NAME`, dataset YAML path `DATASET_YAML_PATH`, training parameters `EPOCHS`, `BATCH_SIZE`, `IMAGE_SIZE`, and `EARLY_STOPPING_PATIENCE`

Output: Training results

```
Step 1: Check local pre-trained weight file;
if MODEL_NAME does not exist then
    Output "Error: Pre-trained weight file not found.";
    Stop execution;

Step 2: Select device;
if GPU is available then
    Use GPU;
else
    Use CPU;

Step 3: Check dataset YAML file;
if DATASET_YAML_PATH does not exist then
    Output "Error: Dataset YAML file not found.";
    Stop execution;

Step 4: Load YOLO model;
Load the model from MODEL_NAME;

Step 5: Output training configuration;
Output training parameters, including EPOCHS, BATCH_SIZE, IMAGE_SIZE, and
  EARLY_STOPPING_PATIENCE;

Step 6: Start training;
Start training the model with the specified parameters and early stopping strategy;
if training is successful then
    Output "Training completed successfully.";
    Return training results;
else
    Output "Error during training.";
```

3 YOLO MODEL PREDICTION AND EVALUATION

Below is the pseudocode describing the core algorithm used for YOLO model prediction and evaluation:

Algorithm 3: YOLO Model Prediction and Evaluation Algorithm

Input: Model path `MODEL_PATH`, test image directory `INPUT_DIR`, label directory `LABELS_DIR`, and ablation mode `ABLATION_MODE`

Output: Evaluation results

Step 1: Load model;

Load the pre-trained YOLO model from `MODEL_PATH`;

Step 2: Initialize output directory;

Create an output directory according to `ABLATION_MODE`;

if *the output directory already exists* **then**

  Delete and recreate the output directory;

Step 3: Process test images;

foreach *image in* `INPUT_DIR` **do**

  Read the image;

  Extract landmarks;

  Predict chromosome instances using `model.predict()`;

  if `ABLATION_MODE` *is* `No_Postprocessing` **then**

    Use the raw output bounding boxes;

  else

    Filter and deduplicate predicted bounding boxes;

  Generate diagnosis labels based on the prediction results;

  Save the processed image with prediction results;

Step 4: Compute evaluation metrics;

Compute the confusion matrix;

Calculate accuracy, sensitivity, and specificity;

Step 5: Output results;

Save the evaluation report in Excel format;

Plot and save the confusion matrix;

Step 6: Completion;

Output final evaluation results;

4 CHROMOSOME STRAIGHTENING ALGORITHM

Below is the pseudocode describing the core algorithm used for chromosome straightening:

Algorithm 4: Chromosome Straightening Algorithm

Input: Input directory `INPUT_DIR`, output directory `OUTPUT_DIR`, and image extensions `image_extensions`
Output: Processed images saved in `OUTPUT_DIR`

Step 1: Batch process images;
Create the output directory if it does not exist;
Get all image files with the specified extensions from `INPUT_DIR`;
Output the total number of images;

Step 2: Process each image;
foreach *image in* `INPUT_DIR` **do**
    Read the image in grayscale format;
    if *the image has a white background with black chromosome regions* **then**
        Invert colors to obtain a black background with white chromosome regions;
    Apply `straighten_chromosome()` to straighten the chromosome;
    Save the processed image to `OUTPUT_DIR`;

Step 3: Straighten chromosome;
Calculate the image size and resize it to the target size while maintaining the aspect ratio;
Extract the middle line of the chromosome from the binarized image;
Apply smoothing to the middle line;
Straighten the image according to the smoothed middle line;
Return the straightened image;

Step 4: Image padding;
Pad the straightened image to the target size 128×256 while maintaining the aspect ratio;
Return the padded image;

5 CHROMOSOME DATA PROCESSING ALGORITHM

Below is the pseudocode describing the core algorithm used for processing chromosome data:

Algorithm 5: Chromosome Data Processing Algorithm

Input: Dataset directory `ROOT_DIR` and save path `SAVE_PATH`
Output: Processed dataset saved in `SAVE_PATH`

```
Step 1: Initialize data structures;
Define a temporary dictionary temp[patient][cell][chrom][side] = seq;
Initialize count = 0;

Step 2: Process dataset files;
foreach image file in ROOT_DIR do
    if the file is not in PNG format then
        Skip this file;
    Parse the filename to extract patient_id, cell_id, chrom_id, and side;
    if side or chrom_id is invalid then
        Skip this file;
    Extract the sequence from the image using extract_sequence_from_image();
    if sequence extraction fails then
        Skip this file;
    Store the extracted sequence in temp[patient_id][cell_id][chrom_id][side];
    Increment count;

Step 3: Pair homologous chromosomes;
foreach patient_id in temp do
    foreach cell_id in temp[patient_id] do
        foreach chrom_id in temp[patient_id][cell_id] do
            if both L and R sequences are available then
                Pair the left and right homologous chromosome sequences;
                Store the paired data in data[patient_id][cell_id][chrom_id];

Step 4: Save processed data;
Save the processed data structure to SAVE_PATH using pickle;

Step 5: Output example;
Print one example from the processed dataset, including patient ID, cell ID, chromosome ID, and paired
  sequence count;
```

6 HOMNET MODEL ALGORITHM

Below is the pseudocode describing the core algorithm used for the HomNet model:

Algorithm 6: HomNet Model Algorithm

Input: Chromosome sequence data `x`, chromosome type index `chrom_idx`, and optional mask `mask`
Output: Classification output `logits`

Step 1: Initialize modules;
Initialize `CMSBlock` for single-chromosome feature extraction;
Initialize `HomBlock` for homologous chromosome difference modeling;
Initialize `chrom_embedding` for chromosome type embedding;
Initialize a fully connected classifier for binary prediction;

Step 2: Process input data;
Reshape input `x` from $(B, Bag, 2, 2, L)$ to $(B \times Bag, 2, 2, L)$;
Separate each homologous chromosome pair into `xa` and `xb`;
Obtain chromosome type embedding `type_emb` from `chrom_idx`;

Step 3: Extract single-chromosome features;
Process `xa` using `CMSBlock` to obtain `feat_a`;
Process `xb` using `CMSBlock` to obtain `feat_b`;

Step 4: Model homologous chromosome differences;
Use `HomBlock` to perform cross-attention-based comparison between `feat_a` and `feat_b`;
Compute the homologous difference representation `diff_vec`;
Fuse `diff_vec` with chromosome type embedding `type_emb`;

Step 5: Aggregate cell-level bag features;
Reshape instance-level features back to (B, Bag, D);
Apply masked max pooling over the bag dimension to obtain `bag_feat`;

Step 6: Compute classification output;
Pass `bag_feat` through the classifier to obtain `logits`;

7 PRETRAINING ALGORITHM

Below is the pseudocode describing the core algorithm used for pretraining the model:

Algorithm 7: Model Pretraining Algorithm

Input: Dataset path `PKL_PATH`, training parameters `EPOCHS`, `BATCH_SIZE`, `LR`, `BAG_SIZE`, `VAL_RATIO`, `ABNORMAL_PROB`, and save directory `SAVE_DIR`
Output: Trained model checkpoints and training logs

```
Step 1: Initialize seed;
Set random seed for reproducibility;
Step 2: Load dataset;
Create the training dataset train_dataset using PretrainChromMIL;
Create the validation dataset val_dataset using PretrainChromMIL;
Step 3: Create data loaders;
Use DataLoader to load train_dataset and val_dataset;
Step 4: Set up model, optimizer, and loss function;
Initialize the HomNet model;
Set the optimizer to Adam;
Set the loss function to CrossEntropyLoss;
Step 5: Train and validate the model;
foreach epoch in EPOCHS do
    Train the model on the training dataset;
    Calculate training loss and training accuracy;
    Validate the model on the validation dataset;
    Calculate validation loss and validation accuracy;
    if validation accuracy improves then
        Save the current model as the best checkpoint;
Step 6: Save model checkpoints;
Save the best model to best_pretrain.pth;
Save the last model to last_pretrain.pth;
Save training logs in JSON format;
Step 7: Visualize training results;
Plot and save training and validation loss curves;
Plot and save training and validation accuracy curves;
Step 8: Completion;
Output final training logs;
Save all logs in JSON format;
```

8 MODEL TRAINING AND EVALUATION ALGORITHM

Below is the pseudocode describing the core algorithm used for model training and evaluation.

Algorithm 8: Model Training Algorithm

Input: Dataset paths `TRAIN_PKL` and `TEST_PKL`, output directory `OUT_DIR`, training parameters `EPOCHS`, `BATCH_SIZE`, and `LR`

Output: Trained model checkpoints and validation results

Step 1: Set random seed;
Set random seed for reproducibility;

Step 2: Load dataset;
Load training and testing data from `TRAIN_PKL` and `TEST_PKL`;
Extract normal and abnormal patient IDs;

Step 3: Configure directories and save paths;
Create a directory for the current run under `OUT_DIR`;

Step 4: Split dataset into training and validation sets;
Use `StratifiedKFold` to split the training set into `N_FOLDS`;
Save training and validation patient IDs for each fold;

Step 5: Define dataset and data loaders;
Use `CellBagDataset` to construct training and validation datasets;
Create training and validation data loaders with `BATCH_SIZE`;
Set class weights and other training parameters;

Step 6: Load pretrained model, if applicable;
if *`USE_PRETRAIN_INIT` is true* **then**
    Load pretrained model weights for fine-tuning;

Step 7: Define optimizer and loss function;
Use the `Adam` optimizer with learning rate `LR`;
Use `CrossEntropyLoss` with class weights;

Step 8: Train model;
foreach *epoch in `EPOCHS`* **do**
    Train the model on the training set;
    Calculate training loss;
    Evaluate the model on the validation set;
    Calculate validation loss and validation accuracy;
    if *validation accuracy improves* **then**
        Save the current model as the best checkpoint;

Step 9: Save model and logs;
Save the best model to `best.pth`;
Save training logs and validation metrics;

Step 10: Output training results;
Output final training and validation results;

Algorithm 9: Threshold Selection and Final Evaluation Algorithm

Input: Best trained model, validation prediction scores, test dataset, and target specificity `TARGET_SPEC`
Output: Final patient-level prediction results and evaluation metrics

Step 1: Compute validation prediction scores;
Apply the trained model to the validation set;
Aggregate cell-level prediction scores into patient-level scores;

Step 2: Choose decision threshold;
Select the optimal threshold using validation results;
Choose the threshold that achieves the target specificity `TARGET_SPEC`;

Step 3: Plot and save validation figures;
Plot and save the ROC curve;
Plot and save the precision–recall curve;
Plot and save the confusion matrix;

Step 4: Perform final evaluation;
Evaluate the trained model on the independent test set;
Aggregate test-set predictions at the patient level;
Apply the selected threshold to obtain final patient-level predictions;

Step 5: Save final results;
Save final prediction scores, binary prediction labels, and evaluation metrics;
Output the final test-set performance results;
